# Chlorhexidine Vs. Sterile Vaginal Wash During Labor to Prevent Neonatal Infection

**DOI:** 10.1155/S1064744997000495

**Published:** 1997

**Authors:** Nancy L. Eriksen, Keri M. Sweeten, Jorge D. Blanco

**Affiliations:** University of Texas Health Science Center—Houston Department of Obstetrics, Gynecology, and Reproductive Sciences Lyndon Baines Johnson Hospital 5656 Kelley Street Houston TX 77026 USA

## Abstract

*Objective:* The purpose of this study was to determine if a dilute solution of chlorhexidine used as a one-time vaginal wash intrapartum can reduce the use of postnatal antibiotics and neonatal infection.

*Methods:* Term pregnant women in labor were prospectively randomized to receive either 20 cc of 0.4% chlorhexidine (n = 481) or 20 cc of sterile water (n = 466) placebo. Exclusion criteria included fetal distress, clinical infection, cervical dilatation >6 cm, and known allergy to chlorhexidine. Outcome variables included the incidence of neonatal pneumonia, culture proven neonatal sepsis, and use of the antibiotics in the neonate. Continuous variables were compared using the Mann-Whitney U-test and discrete variables were compared with the chi-square test.

*Results:* The length of ruptured membranes (mean ± S.D.) between the chlorhexidine group (408 ± 589 min) and control group (352 ± 318 min) was not significantly different (*P* = 0.85, 95% confidence interval 354–462). Fifteen neonates (3.2%) in the chlorhexidine group and 9 (1.9%) in the control group received antibiotics in the postnatal period (*P* = 0.32, 95% confidence interval 0.72–3.72). There was one case of pneumonia in the control group and no cases of sepsis in either group.

*Conclusions:* A one-time chlorhexidine vaginal wash does not decrease the use of antibiotics or incidence of neonatal infection in our population.


Infectious Diseases in Obstetrics and Gynecology 5:286–290 (1997)
© 1998 Wiley-Liss, Inc.

### Chlorhexidine Vs. Sterile Vaginal Wash During Labor to Prevent Neonatal Infection

Nancy L. Eriksen,* Keri M. Sweeten, and Jorge D. Blanco
*University of Texas Health Science Center, Department of Obstetrics, Gynecology, and Reproductive Sciences, Lyndon Baines Johnson Hospital, Houston, TX*

### Abstract

*Objective:* The purpose of this study was to determine if a dilute solution of chlorhexidine used as a one-time vaginal wash intrapartum can reduce the use of postnatal antibiotics and neonatal infection.

*Methods:* Term pregnant women in labor were prospectively randomized to receive either 20 cc of 0.4% chlorhexidine (n = 481) or 20 cc of sterile water (n = 466) placebo. Exclusion criteria included fetal distress, clinical infection, cervical dilatation >6 cm, and known allergy to chlorhexidine. Outcome variables included the incidence of neonatal pneumonia, culture proven neonatal sepsis, and use of the antibiotics in the neonate. Continuous variables were compared using the Mann-Whitney U-test and discrete variables were compared with the chi-square test.

*Results:* The length of ruptured membranes (mean ± S.D.) between the chlorhexidine group (408 ± 589 min) and control group (352 ± 318 min) was not significantly different ($P$ = 0.85, 95% confidence interval 354–462). Fifteen neonates (3.2%) in the chlorhexidine group and 9 (1.9%) in the control group received antibiotics in the postnatal period ($P$ = 0.32, 95% confidence interval 0.72–3.72). There was one case of pneumonia in the control group and no cases of sepsis in either group.

*Conclusions:* A one-time chlorhexidine vaginal wash does not decrease the use of antibiotics or incidence of neonatal infection in our population. Infect. Dis. Obstet. Gynecol. 5:286–290, 1997.
© 1998 Wiley-Liss, Inc.

Key words
neonatal sepsis; intraamniotic infection; vaginal irrigation

Neonatal infections, particularly following the diagnosis of intrapartum infection, result in significant morbidity each year. Infants diagnosed with probable sepsis have significantly longer hospital stays and use of antibiotics leading to increasing hospital costs.

More than a decade ago investigators proposed antepartum vaginal washing with chlorhexidine as a means of decreasing group B streptococcus (GBS) colonization rates in neonates.[1–3] Chlorhexidine disinfection during labor is a simple, cheap, and safe method of reducing GBS colonization without risk for development of bacterial resistance. Another advantage is that it not only can be used in modern labor and delivery suites, but also in the developing world.

Intrigued by these initial results and the possibility that chlorhexidine might also reduce the transmission of other potential pathogens, we postulated that this approach may decrease infectious morbidity in neonates caused by vaginal microorganisms. We have previously reported our maternal data.[4] Therefore, this study sought to determine if a chlorhexidine vaginal wash given intrapartum can

*Correspondence to: Dr. Nancy L. Eriksen, University of Texas Health Science Center—Houston, Department of Obstetrics, Gynecology, and Reproductive Sciences, Lyndon Baines Johnson Hospital, 5656 Kelley Street, Houston, TX 77026.

Received 27 March 1997
Accepted 22 September 1997

reduce the use of antibiotics and infectious morbidity in the neonate.

### SUBJECTS AND METHODS

This randomized control trial was approved by the Committee for the Protection for Human Subjects at the University of Texas Health Science Center—Houston. Women admitted to the Lyndon Baines Johnson Hospital labor and delivery room between June 1991 through September 1992 were considered for enrollment. Patients at 36 weeks or greater gestational age in labor were counseled about the study and were invited to participate. Exclusion criteria on admission to the hospital included preterm labor, fetal distress, malpresentation, intraamniotic infection, cervical dilatation >6 cm, and known allergy to chlorhexidine. All patients meeting eligibility criteria were invited to participate and written informed consent was obtained from all participating women.

A computer software program (True Epistat, Richardson, TX) was used to generate a random block allocation sequence to assign patients to either group. The randomization assignments were contained in sequentially numbered, opaque, sealed packets that were made up independent of the physicians managing the patients. The randomization was known only after the patient had been enrolled in the study. The authors chose not to blind the study because the syringe containing the chlorhexidine solution was pink and we could not reproduce the color artificially in the syringe containing the sterile water. Additionally, the physicians managing infants in the nursery were unaware of which arm of the study each patient was randomized, thus eliminating physician bias.

Patients presenting to labor and delivery who did not have a history of rupture of the membranes underwent a vaginal examination prior to inclusion in the study. Women who had documented rupture of the membranes had their cervical dilatation determined by visual examination of the cervix with a speculum. Following enrollment all patients underwent a speculum examination by the admitting resident physician. Women randomized to the study arm received 20 cc of a 0.4% chlorhexidine solution. The solution was placed around the portio and fornices using a syringe. Women in the control group were irrigated with 20 cc of sterile water. Clinical information was abstracted from the medical record by a study nurse including demographic information, labor characteristics, the use of antibiotics in the neonatal period (primary outcome), and the incidence of neonatal infection. The data were collected after all neonates had been discharged from the hospital. The investigators did not know the clinical outcome of the infants prior to the end of the study.

The clinical diagnosis of intraamniotic infection (IAI) was made when the patient had an intrapartum temperature ≥100.4°F with two of the following criteria: maternal tachycardia, uterine tenderness, foul amniotic fluid, material leukocytosis, or fetal tachycardia. In nearly all cases, initial therapy consisted of parental ampicillin plus gentamicin.

A diagnosis of neonatal infection was made if the neonate developed pneumonia or sepsis. The diagnosis of neonatal pneumonia was made by the attending physician if the neonate was febrile and had chest radiograph findings consistent with the diagnosis. Neonatal sepsis was diagnosed if the infant had a positive blood or cerebrospinal fluid culture, along with a clinical course consistent with sepsis.

A sample size calculation was performed based on a prestudy neonatal antibiotic use rate of 6%. In order to reduce the use of antibiotics to 3%, it would require 744 patients, enrolled in each arm in the study in order to have an 80% power with a $P < 0.05$. During the study period we observed a declining IAI rate in our hospital and subsequent decline in the use of antibiotics in the neonatal period, therefore, we performed an interim analysis. Statistical analysis was performed using a computer software package (True Epistat). Tests for normality showed that the data were not normally distributed. Continuous data were analyzed using the Mann-Whitney U-test. Categorical data were analyzed with the chi-square test. $P < 0.05$ was considered statistically significant.

### RESULTS

Informed consent was obtained on 1,024 women who were eligible for the study. Of these, 77 were excluded from the analysis for the following reasons: incomplete records (71), the patient was enrolled and subsequently discharged home (3), vaginal wash was not given (2), and a patient whose infant had anencephaly was inadvertently enrolled

TABLE 1. Demographic data[a]

| Characteristic | Chlorhexidine (n = 457) | Control (n = 453) | P |
|---|---|---|---|
| Estimated gestational age (weeks) | 39.4 ± 1.5 | 39.3 ± 2.4 | 0.69 |
| Birth weight (g) | 3,369 ± 449 | 3,365 ± 447 | 0.75 |
| 5 min Apgar <7 | 4 (0.9%) | 6 (1.3%) | 0.75 |
| Days in hospital | 2.2 ± 2.1 | 2.2 ± 2.2 | 0.40 |

[a]Data are expressed as mean ± S.D., except for 5 min Apgar <7 data, which are expressed as number (%).

(1). Of the remaining 947 patients, 481 were randomized to the study arm and 466 served as controls. Twenty-four neonatal charts in the chlorhexidine group and 13 in the control group were unavailable for review. This left 457 neonates in the study group and 453 in a control group for analysis. Demographic data are expressed in Table 1. The two groups were similar with respect to estimated gestational age, birth weight, Apgar score <7 at 5 min, and the number of days in the hospital.

Fifteen (3.2%) neonates in the chlorhexidine group and 9 (1.9%) in the control group received antibiotics ($P = 0.32$, 95% confidence interval 0.72–3.72). There was one case of pneumonia in the control group and no cases of neonatal sepsis in either group.

Patients were also analyzed for any relationship between labor characteristics and use of antibiotics in the neonate and the presence or absence of IAI (Table 2). Patients with a diagnosis of IAI in either group had a significantly longer duration of ruptured membranes compared to women without IAI. The time between the vaginal wash and delivery was significantly greater in patients with IAI compared to those without infection in the chlorhexidine group but not for controls. The number of neonates receiving antibiotics in either group was significantly greater if the mother had a diagnosis of IAI.

No adverse effects of chlorhexidine prophylaxis were observed among any neonate.

### DISCUSSION

Chlorhexidine gluconate is a potent antimicrobial agent with activity against vaginal bacteria causing neonatal infection.[5] It can suppress growth of bacteria for up to 24 h and its effectiveness is not reduced by the presence of blood or amniotic fluid.[1,6]

Chlorhexidine applied to the vagina intrapartum has been shown to decrease the transmission of GBS to neonates and reduce the GBS colonization of women in the puerperium.[1] Chlorhexidine has also been used as a skin and mucous membrane disinfectant in nurseries for many years. Absorption of chlorhexidine from intact skin is negligible and there is virtually no acute toxicity in animal studies.[7] Concentrations of up to 8% have been used for daily bathing of neonatal monkeys as a means to provide additional evidence of its safety as a skin cleanser.[8] Other studies show that absorption of chlorhexidine through intact skin into the venous blood is negligible.[9] Therefore, chlorhexidine vaginal wash given intrapartum appears to be an inexpensive and safe antimicrobial method for potentially reducing peripartum and neonatal infectious morbidity.

The results of this study indicate that there is no difference in the incidence in the use of antibiotics in the neonatal period or the incidence of neonatal infection. The use of antibiotics appeared to correlate with the length of ruptured membranes as well as presence of IAI. Both of these are known risk factors for neonatal sepsis and pneumonia.[10] In a similar study to ours, Rouse and co-workers[11] showed no difference in neonatal outcomes, including sepsis.

Several reasons could explain our findings. Perhaps, a 10-fold dilution of the stock solution (4%) is not an effective antimicrobial strength. Although this possibility exits, the chlorhexidine concentration used in our study was 300 times the minimum bactericidal concentration for the more common neonatal pathogens. Furthermore, the concentration used in our study was somewhat higher than what has been used in other clinical studies.[1–3]

Another explanation for the lack of any difference between groups is the vehicle used. An aqueous chlorhexidine solution has been used extensively by Christensen's group.[2] Perhaps another vehicle with a higher viscosity, such as a gel, might be more adherent to the vaginal walls and thus increase its effectiveness when compared to an aqueous solution. Recently, these two methods were compared. Although there was a tendency toward more full-term neonates with sepsis in a group using chlorhexidine gel, there was no differ-

TABLE 2. Labor characteristics and use of antibiotics in the neonate compared to the incidence of IAI[a]

| | Chlorhexidine | | | Controls | | |
|---|---|---|---|---|---|---|
| Characteristic | IAI (n = 23) | No IAI n = 434) | *P* | IAI (n = 21) | No IAI (n = 445) | *P* |
| Length of ruptured membranes (min) | 653 ± 408 | 405 ± 540 | <0.001 | 560 ± 373 | 350 ± 321 | 0.005 |
| Vaginal wash to delivery (min) | 559 ± 235 | 326 ± 274 | <0.001 | 433 ± 364 | 312 ± 260 | NS |
| Antibiotics | 8 (35%) | 7 (1.6%) | <0.001 | 6 (29%) | 3 (0.7%) | <0.001 |

[a]Data are expressed as mean ± S.D., except for antibiotic data, which are expressed as number (%). NS = not significant.

ence in the number of neonates receiving systemic antibiotics between the group receiving aqueous chlorhexidine compared to chlorhexidine gel.[12]

On the other hand, Christensen's group[2] described a complex, vigorous, extensive washing procedure of the cervix, vagina, and external genital. A beneficial effect might be ascribed to both the mechanical cleansing effect and the antimicrobial action of the chlorhexidine.

There is also the possibility of a significant washout effect of the bacteria in the placebo group receiving normal saline. Stray-Pedersen et al. (unpublished abstract, XIII World Congress of Gynecology and Obstetrics, 1991) demonstrated a significant reduction in the transmission rate of microbes in patients receiving saline or chlorhexidine douche compared to women who did not receive a douche intrapartum.

In our trial, we chose to use a single vaginal application as others have described.[1–3,11] Other studies, analyzing the effects of vaginal washing on neonatal morbidity, repeated the wash (every 6 h) until delivery.[13] While future studies may later prove this to be more beneficial, it is not practical at our institution, since the mean time from wash to delivery was just over 5 h.

While any study that does not demonstrate a statistically significant difference may have a type II error, our data have an 80% power to detect a 4% difference in the use of systemic antibiotics in the neonate. Thus, it is possible our sample size was too small to detect a small difference in antibiotic use that usually exists. Using the observed use of antibiotics in our control group (1.9%) we would need to enroll 2,649 patients in each arm in order to have an 80% power. Given the magnitude of such a study, it would not be feasible to accomplish.

Our data show that a one-time vaginal wash with chlorhexidine does not improve neonatal outcome. Further studies using repeated washes, mechanical cleansing, or a different vehicle may be necessary in order to demonstrate any difference in neonatal outcome.

### REFERENCES

1. Dykes A, Christensen K, Christensen P, Kahlmeter G: Chlorhexidine for prevention of neonatal colonization with group B streptococci II. Chlorhexidine concentrations and recovery of group B streptococci following vaginal washings in pregnant women. Eur J Obstet Gynecol Reprod Biol 16:167–172, 1983.
2. Christensen K, Christensen P, Dykes A, Kahlmeter G: Chlorhexidine for prevention of neonatal colonization with group B streptococci. III. Effect of vaginal washing with chlorhexidine before rupture of the membranes. Eur J Obstet Gynecol Reprod Biol 19:231–236, 1985.
3. Kellee LA, Speyer I, vanKuijck MA, Koopman R, Dony JM, Bakker JH, et al.: Prevention of group B streptococci transmission during delivery by vaginal application of chlorhexidine gel. Eur J Obstet Gynecol Reprod Biol 31:47–51, 1989.
4. Sweeten KM, Eriksen NL, Blanco JD: Chlorhexidine versus sterile water vaginal wash during labor to prevent peripartum infection. Am J Obstet Gynecol 176:426–430, 1997.
5. Vorherr H, Vorherr UF, Mehta P, Ulrich JA, Messer RH: Antimicrobial effect of chlorhexidine and providing-iodine on vaginal bacteria. J Infect 8:195–199, 1984.
6. Christensen K, Christensen P, Dykes AK, Kahlmeter G, Kurl D, Linden V: Chlorhexidine for prevention of neonatal colonization with group B streptococci I. In vitro effect of chlorhexidine on group B streptococci. Eur J Obstet Gynecol Reprod Biol 16:157–165, 1983.
7. Case DE, McAnish J, Rushton A: Chlorhexidine: Attempts to detect percutaneous absorption in man. Proc 9th Int Cong Chemother 3:367–374, 1976.
8. Gongwer LE, Hubben K, Lenkiewicz RS, Hart ER, Cockrell BY: The effects of daily bathing of neonatal rhesus monkeys with an antimicrobial skin cleanser containing chlorhexidine gluconate. Toxicol Appl Pharmacol 52:255–261, 1980.
9. Cowen J, Ellis SH, McAnish J: Absorption of chlorhexidine from the intact skin of newborn infants. Arch Dis Child 54:379–383, 1979.

10. Hauth JC, Gilstrap LC, Hankins GDV, Connor KD: Term maternal and neonatal complications of acute chorioamnionitis. Obstet Gynecol 66:59–62, 1985.
11. Rouse DJ, Hauth JC, Andrews WW, Mills BB, Maker JE: Chlorhexidine vaginal irrigation for the prevention of peripartal infection: A placebo-controlled randomized clinical trial. Am J Obstet Gynecol 176:617–622, 1997.
12. Henrichsen T, Lindemann R, Svenningsen L, Hjelle K: Prevention of neonatal infections by vaginal chlorhexidine disinfection during labor. Acta Pediatr 83:923–926, 1994.
13. Burman LG, Christensen P, Christensen K, Fryklund B, Helgesson A, Svenningsen NW, Tullus K. Prevention of excess neonatal morbidity associated with group B streptococci by vaginal chlorhexidine disinfection during labor. Lancet 340:65–69, 1992.
